# Extrinsic Protein Tyrosine Phosphatase Non-Receptor 22 Signals Contribute to CD8 T Cell Exhaustion and Promote Persistence of Chronic Lymphocytic Choriomeningitis Virus Infection

**DOI:** 10.3389/fimmu.2017.00811

**Published:** 2017-07-12

**Authors:** Tatiana Jofra, Giuseppe Galvani, Mirela Kuka, Roberta Di Fonte, Bechara G. Mfarrej, Matteo Iannacone, Shahram Salek-Ardakani, Manuela Battaglia, Georgia Fousteri

**Affiliations:** ^1^Division of Immunology Transplantation and Infectious Diseases (DITID), Diabetes Research Institute (DRI) IRCCS San Raffaele Scientific Institute, Milan, Italy; ^2^Department of Pathology, Immunology and Laboratory Medicine, University of Florida, Gainesville, FL, United States

**Keywords:** protein tyrosine phosphatase non-receptor 22, persistent viral infection, T cell exhaustion, lymphocytic choriomeningitis virus clone 13, genetic predisposition

## Abstract

A genetic variant of the protein tyrosine phosphatase non-receptor 22 (PTPN22) is associated with a wide range of autoimmune diseases; however, the reasons behind its prevalence in the general population remain not completely understood. Recent evidence highlights an important role of autoimmune susceptibility genetic variants in conferring resistance against certain pathogens. In this study, we examined the role of PTPN22 in persistent infection in mice lacking PTPN22 infected with lymphocytic choriomeningitis virus clone 13. We found that lack of PTPN22 in mice resulted in viral clearance 30 days after infection, which was reflected in their reduced weight loss and overall improved health. PTPN22^−/−^ mice exhibited enhanced virus-specific CD8 and CD4 T cell numbers and functionality and reduced exhausted phenotype. Moreover, mixed bone marrow chimera studies demonstrated no differences in virus-specific CD8 T cell accumulation and function between the PTPN22^+/+^ and PTPN22^−/−^ compartments, showing that the effects of PTPN22 on CD8 T cells are T cell-extrinsic. Together, these findings identify a CD8 T cell-extrinsic role for PTPN22 in weakening early CD8 T cell responses to collectively promote persistence of a chronic viral infection.

## Introduction

The autoimmune predisposing allele of protein tyrosine phosphatase non-receptor 22 (PTPN22) has attracted a lot of attention due to its association with several autoimmune diseases such as type 1 diabetes and rheumatoid arthritis (1, 2). Studies in mouse models of autoimmunity have shown that PTPN22 can have an inhibitory or accelerating influence on disease progression that is dependent on the genetic background, the overall immunological context, and importantly, the type of effect on the disease-driving or regulatory T cell subset (3). PTPN22 plays a dual role in the immune system; on the one hand, it dampens effector T cell responses, but on the other hand, it promotes the activation and pro-inflammatory cytokine production by innate immune cells (4). Likely, this dual role of *Ptpn22* in innate and adaptive immunity is responsible for the confounding results observed in the different disease models studied so far.

It has been puzzling how a gene that has such a strong impact on autoimmunity would show high prevalence in the general population. Recent evidence suggests that many of the genes conferring susceptibility to autoimmunity control immunoregulatory factors that are implicated in host–pathogen interactions (5). Thus, it is possible that the *Ptpn22* autoimmune predisposing allele provides an immunological advantage. Interestingly, several of the phenotypes described in mice knocked-in with the human autoimmune *Ptpn22* allele are similar to those seen in *Ptpn22*-deficient mice (6–8). Recent studies addressing the role of *Ptpn22* in antiviral immunity either by studying PTPN22^−/−^ mice or cells from carriers of the autoimmune-associated variant showed a clear link between PTPN22 and effective responses to acute viral infection (9). In the setting of acute lymphocytic choriomeningitis virus Armstrong (LCMV Arm) infection, it has been shown that mice deficient for PTPN22 produce reduced number of LCMV-specific cytotoxic lymphocytes (CTLs) due to a weakened innate immune response and T cell-intrinsic defects in response to type I IFNs (10–12). However, pathogen control is largely unaltered, suggesting that the role of PTPN22 in the setting of acute LCMV Arm infection is largely dispensable (11).

The dual function of PTPN22 in innate and adaptive immunity might be reflected in the T cell functional or developmental phenomena that occur during viral persistence. Hence, we tested the biological relevance of PTPN22 in the lymphocytic choriomeningitis virus clone 13 (LCMV Cl13) viral infection system, where two amino acid substitutions in the virus genome cause persistent infection for more than 3 months, leading to antiviral CD4 and CD8 T cells that are physically deleted or persist in a “non-functional” state and characterized by the inability to produce effector cytokines (exhausted T cells) (13–16). We show that mice deficient for PTPN22 are able to control the virus 30 days after infection, show reduced weight loss, and generate higher numbers of LCMV-specific CTLs and CD4 T cells with heightened function. To address whether the increased exhaustion of T cells in PTPN22^+/+^ mice was due to direct effects of PTPN22 on T cells or on another cell type that changes viral load and therefore the degree of exhaustion, we performed mixed bone marrow chimeras. These experiments showed that the effects of PTPN22 on CD8 T cells are largely cell-extrinsic. Taken together, our studies provide evidence for a critical role for PTPN22 in suppressing the initial priming and functional differentiation of CD8 T cells to promote viral persistence.

## Materials and Methods

### Mice, Virus, and Infection

Wild-type and *Ptpn22*-deficient (PTPN22^−/−^) littermate mice were used (6). Mice were housed under specific pathogen free conditions in compliance with the guidelines of the San Raffaele Institutional Animal Care and Use Committee (approved protocol #677). LCMV Cl13 plaque-purified was prepared by a single passage on BHK-21 cells and used throughout the experiments. A single dose of 2 × 10^6^ PFU i.v. was used to induce chronic infection as previously described (17).

### Virus Titration

Lymphocytic choriomeningitis virus titers of infected spleens were determined in a virus plaque assay as previously described (11). Tissue samples (frozen at −80°C) were thawed, homogenized in MEM, and added to the MC57 cells in fivefold dilution steps. 4 h later, methylcellulose (Methocel, Sigma, MO, USA) was diluted 1:2 in 2× Dulbecco-modified Eagle’s medium and added to the wells. After 48 h of incubation, cell monolayers were fixed with 4% formaldehyde and permeabilized by incubation in 0.5% Triton X-100. Plaques of virus-infected cell clusters were detected by IHC using the LCMV-specific anti-VL-4 mAb. Peroxidase-coupled anti-rat Ig antibody (Jackson ImmunoResearch, PA, USA) was used as a secondary antibody, and a color reaction was performed using 3,3′-diaminobenzidine tablets (SIGMAFAST™ Sigma, MO, USA).

### Mixed Bone Marrow Chimeras

PTPN22^+/+^ (CD45.1^−^CD45.2^+^):PTPN22^−/−^ (CD45.1^+^CD45.2^+^) mixed bone marrow chimeras were generated by i.v. reconstituting lethally irradiated C57BL/6 mice (CD45.1^+^CD45.2^−^) with a 1:1 mixture of bone marrow cells for a total of 10 × 10^6^ cells. For control, PTPN22^+/+^ (CD45.1^−^CD45.2^+^) (littermates of PTPN22^−/−^ mice):PTPN22^+/+^ (CD45.1^+^CD45.2^+^) mixed bone marrow chimeras were generated. Recipients were treated with gentamycin 400 mg/l starting 1 week prior to reconstitution until 2 weeks after reconstitution.

### Flow Cytometry

Following an Fc (anti-CD16/32) blocking step, cells were stained with anti-CD3, -CD4, -CD8, -CD45.1, -CD45.2, -CD25, -CD62L, -CD44, -CD127, -KLRG1, -CD19, -B220, -PD-1, -LAG-3, -LFA-1, -SLAM, -CXCR5, -ICOS, -IgD, -CD138, and -GL7 mAbs (Miltenyi, BD, Biolegend and eBioscience), and then intracellularly with anti-T-BET and -IRF4 mAb using the FOXP3 Fixation/Permeabilization kit (eBioscience). D^b^GP_33–41_ and D^b^NP_396–404_ MHC class I dextramers (DMRs) were used (Immudex) as described previously (11). Following *in vitro* stimulation for 3 h with 1 µg/ml of the class I peptides (GP_33–41_, NP_396–404_, GP_276–286_, and NP_205–212_) and 4 h with 2 µg/ml of the class II epitope (GP_61–80_) in the presence of brefeldin A, cells were stained for IFN-γ, TNF, and IL-2 using the Cytofix/Cytoperm kit (BD) according to manufacturer’s instructions. All samples were acquired on a FACSCanto flow cytometer (BD).

### Statistical Analysis

Comparisons between groups were performed Kruskal–Wallis analysis followed by Dunn’s posttest between groups without correction. Differences between PTPN22^+/+^ and PTPN22^−/−^ were also analyzed with the Mann–Whitney test. For weight loss, the log rank test was applied. In all cases, the Prism software (GraphPad, USA) was used. All error bars indicate SEM. Asterisks in the graphs indicate significant *p* values as follows: *p < 0.05, **p < 0.01, ***p < 0.001.

## Results

### Improved Viral Control in the Absence of PTPN22

**Figure 1:**
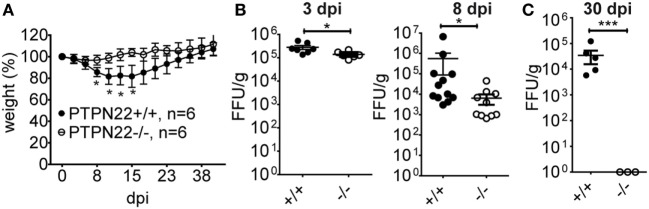
PTPN22^−/−^ mice show enhanced control of chronic lymphocytic choriomeningitis virus clone 13 (LCMV Cl13) infection. PTPN22^+/+^ (black circles) and PTPN22^−/−^ (white circles) mice were infected with 2 × 10^6^ PFU of LCMV Cl13 i.v. **(A)** Body weight was measured twice weekly and the degree of weight loss served as a marker of disease severity. **(B,C)** Spleens were harvested after 3, 8, and 30 days post infection (dpi), and viral loads were determined by focus assay (focus forming unit, FFU). Data in **(A)** are representative of three experiments with similar results. Data in **(B,C)** are pooled from two experiments with similar results. Number of symbols reflects number of mice per group.

To analyze the effect of PTPN22 on T cell exhaustion and virus clearance in chronic LCMV Cl13 infection, we infected PTPN22^+/+^ and PTPN22^−/−^ mice with 2 × 10^6^ PFU LCMV Cl13. Because LCMV Cl13 infection causes severe immunopathology characterized by extensive weight loss that in some cases leads to severe comorbidity (18), we monitored weight loss over time. Interestingly, PTPN22^−/–^ mice had a much healthier appearance that was reflected in their reduced weight loss (Figure 1A). Next, we analyzed whether PTPN22 affects the capability of mice to control viral replication. We quantified viral titers in the spleen at various time points after infection. Strikingly, we found that PTPN22^−/−^ mice were characterized by lower viral titers as early as 3 and 8 days post infection (dpi) (Figure 1B). This difference was even more remarkable by 30 dpi, when viral titers dropped below the detection limit in the spleens of PTPN22^−/−^ mice, while PTPN22^+/+^ mice displayed consistently higher viral titers (Figure 1C).

### Increased Quantity and Quality of LCMV-Specific CTLs in the Absence of PTPN22

**Figure 2:**
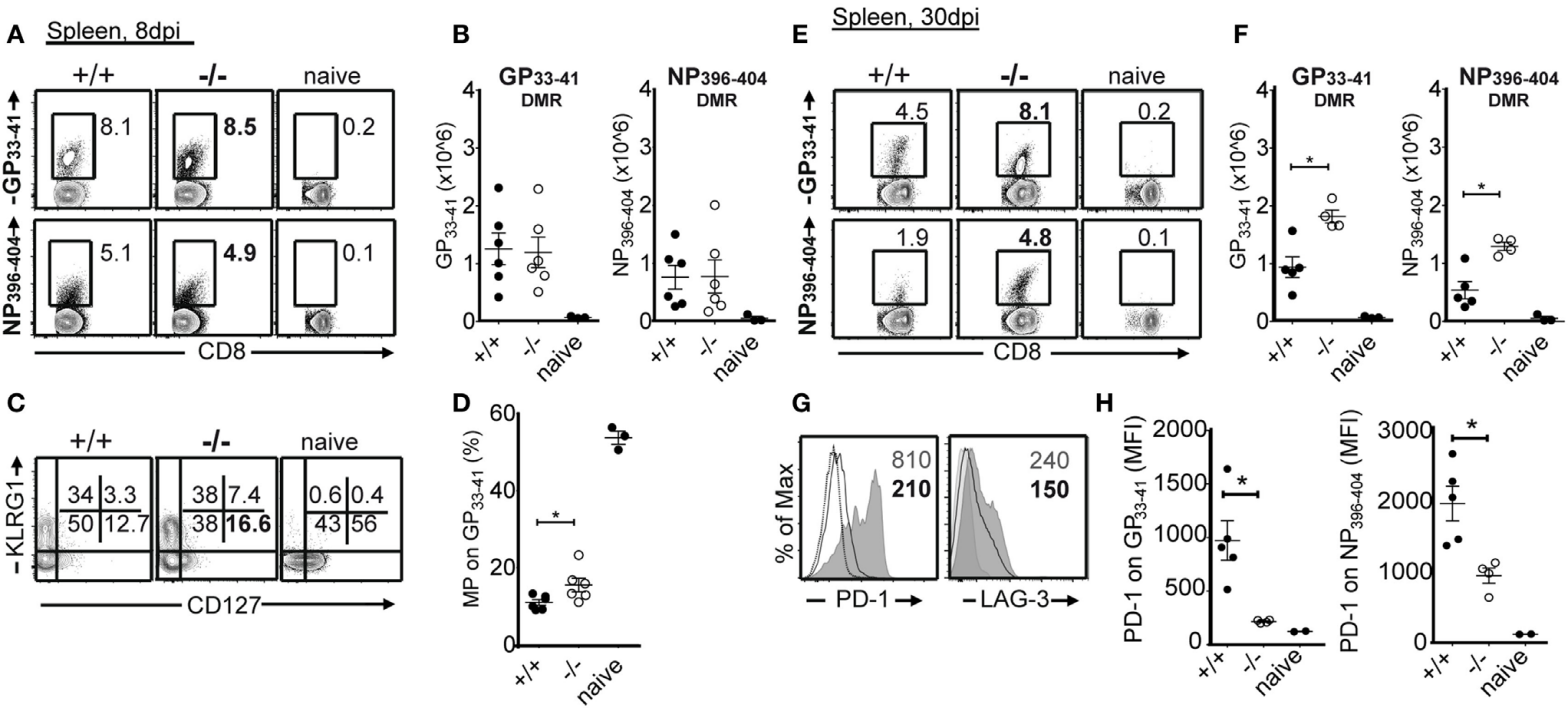
PTPN22^−/−^ mice exhibit enhanced virus-specific CD8 T cell numbers and reduced exhaustion phenotype. PTPN22^+/+^ (black circles) and PTPN22^−/−^ (white circles) mice were infected as described in Figure 1. **(A,B)** Virus-specific splenic T cell populations were analyzed 8 dpi using GP_33–41_ and NP_396–404_ dextramers (DMRs) (gate: CD3^+^B220^−^CD8^+^CD44^+^). **(C,D)** CD127 and KLRG1 costaining was used to distinguish terminal effector (CD127^−^KLRG1^+^) and memory precursor (CD127^+^KLRG1^−^) GP_33–41_^+^ cells. **(E,F)** Virus-specific splenic T cell populations were analyzed 30 dpi using GP_33–41_ and NP_396–404_ DMRs (gate: CD3^+^B220^−^CD8^+^). **(G,H)** PD-1 and LAG-3 expression levels (geometric mean) were analyzed on GP_33–41_^+^ cells from representative PTPN22^+/+^ (gray filled histogram) and PTPN22^−/−^ (black solid line) mice 30 dpi. Naïve CD8 T cells (black dotted line) were used as control. Representative contour plots or histogram overlays show one mouse per group. Data are derived from a total of three independent experiments, one representative experiment is shown per graph. Number of symbols reflects number of mice per group.

Because LCMV Cl13-infected mice experience severe lymphopenia caused by immunopathology, we analyzed T and B cell populations 8, 15, and 30 dpi in PTPN22^+/+^ and PTPN22^−/−^ animals. We found that the absence of PTPN22 did not alter LCMV Cl13-induced lymphopenia (data not shown). Next, we analyzed the impact of PTPN22 on LCMV-specific T cell responses. Using MHC class I-restricted DMRs, we detected similar frequency and number of LCMV-specific (GP_33–41_ and NP_396–404_) CD8 T cells in PTPN22^−/−^ and PTPN22^+/+^ mice 8 dpi (Figures 2A,B). Whereas the proportion of terminal effector (CD127^−^KLRG1^+^) was similar within LCMV-specific CTLs, memory precursor cells (CD127^+^KLRG1^−^) were increased in PTPN22^−/−^-infected animals (Figures 2C,D). Frequency and number of LCMV-specific CTLs was higher in PTPN22^−/−^ mice 30 dpi (Figures 2E,F), showing that in PTPN22^−/−^ mice where the virus is cleared faster, cells could differentiate earlier into memory cells. The expression levels (geometric mean) of PD-1 and LAG-3, two molecules that are highly upregulated on exhausted T cells (19), were considerably lower on LCMV-specific CTLs from PTPN22^−/−^ mice 30 dpi (Figures 2G,H).

**Figure 3:**
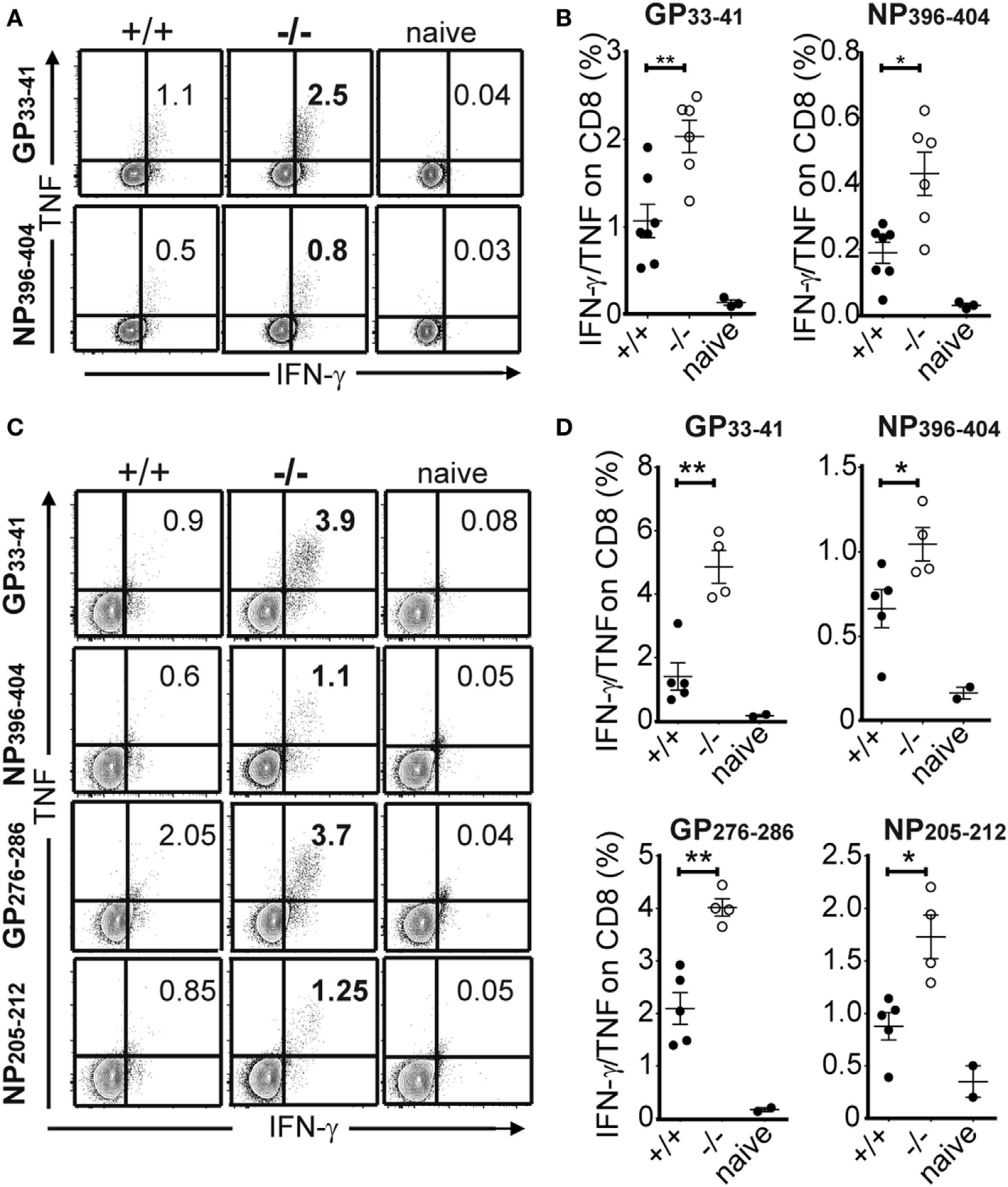
PTPN22^−/−^ mice show improved antiviral cytotoxic lymphocyte function. PTPN22^+/+^ (black circles) and PTPN22^−/−^ (white circles) mice were infected as described in Figure 1. **(A–D)** intracellular cytokine staining was performed following *in vitro* stimulation with immunodominant CD8 epitopes at 8 **(A,B)** and 30 dpi **(C,D)**. IFN-γ/TNF-α double producing cells are depicted in the plots. Data are derived from a total of three independent experiments, one representative experiment is shown per graph. Number of symbols reflects number of mice per group.

**Figure 4:**
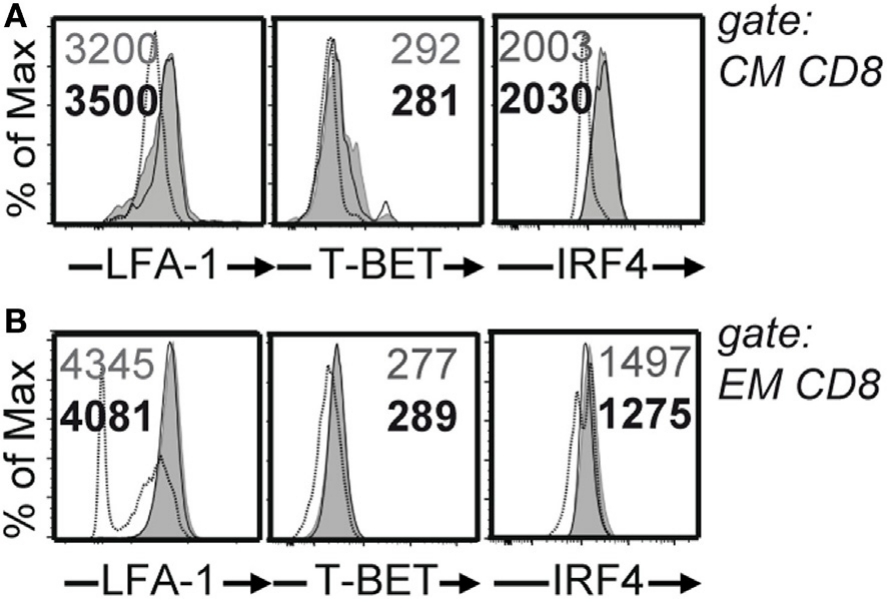
Similar levels of LFA-1, T-BET, and IRF4 on CD8 T cells in the absence of protein tyrosine phosphatase non-receptor 22 (PTPN22). PTPN22^+/+^ (black circles) and PTPN22^−/−^ (white circles) mice were infected as in Figure 1. **(A,B)** LFA-1, T-BET, and IRF4 levels were determined in PTPN22^+/+^ and PTPN22^−/−^ central memory (CD44^+^CD62L^+^) **(A)** and effector memory (CD44^+^CD62L^−^) **(B)** polyclonal CD8 cells 8 days post infection (dpi). Representative histogram overlays show one mouse per group. Data are derived from a total of three independent experiments, one representative experiment is shown per graph.

The magnitude and functionality of the anti-LCMV CD8 T cell responses were then assessed by epitope-specific intracellular cytokine staining. By analyzing the expression of key effector and stimulatory cytokines, IFN-γ, TNF, and IL-2 (19), we found greater numbers of cytokine producing cells in PTPN22^−/−^ animals in response to class I epitopes as compared to controls at 8 and 30 dpi (Figure 3). We characterized the expression of LFA-1, T-BET, and IRF4, whose expression levels associate with effector function (20–22). No differences were observed among PTPN22^+/+^ and PTPN22^−/−^ central memory-like (CD44^+^CD62L^+^) and effector memory-like (CD44^+^CD62L^−^) polyclonal CTLs 8 dpi (Figure 4). Together, these findings reveal an important role for PTPN22 in the CD8 T cell response and control of a chronic LCMV infection.

### PTPN22 Controls Antiviral CD4 T Cell Responses to LCMV Cl13

**Figure 5:**
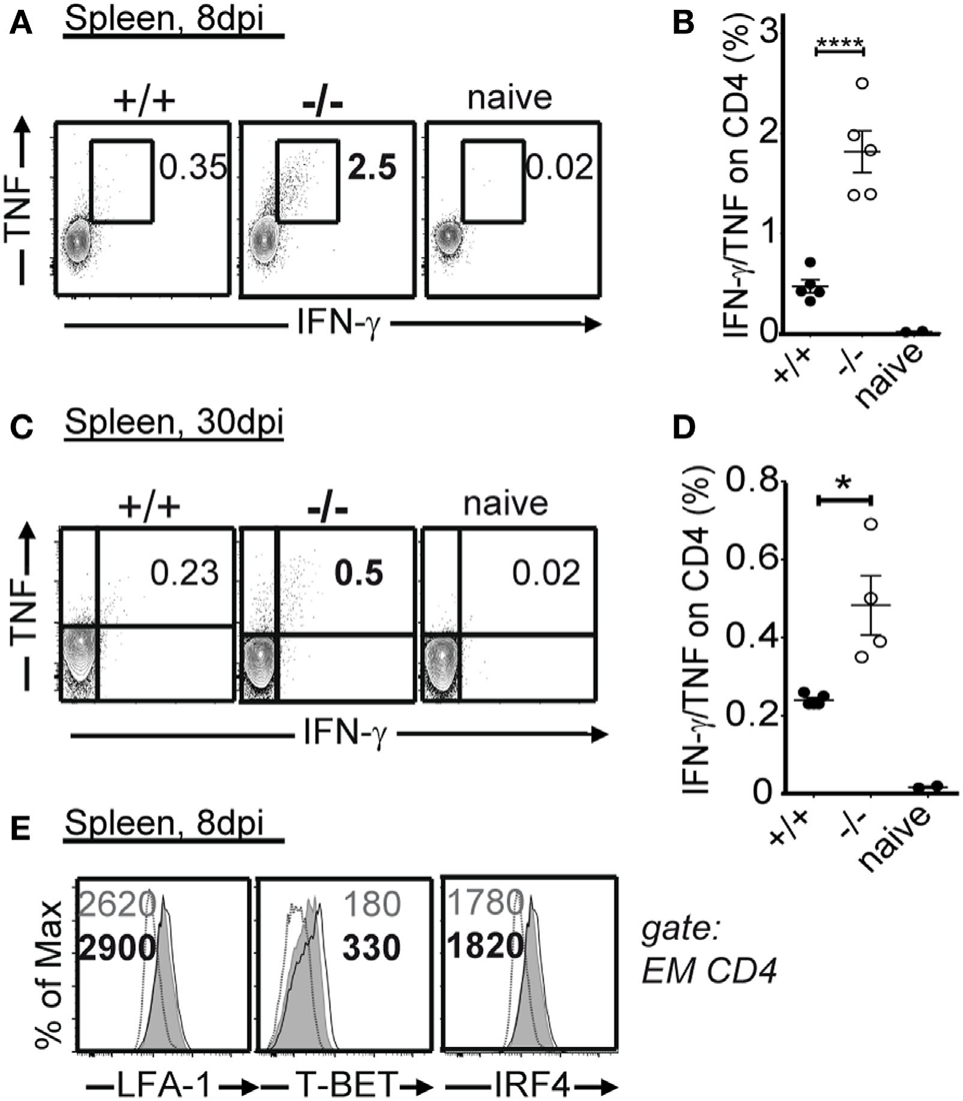
PTPN22^−/−^ mice have improved antiviral CD4 T responses following lymphocytic choriomeningitis virus clone 13 infection. PTPN22^+/+^ (black circles) and PTPN22^−/−^ (white circles) mice were infected as in Figure 1. **(A–D)** Production of IFN-γ and TNF by GP_61–80_-specific CD4 T cells was performed by intracellular cytokine staining at 8 **(A,B)** and 30 days post infection (dpi) **(C,D)**. **(E)** LFA-1, T-BET, and IRF4 levels were determined in PTPN22^+/+^ and PTPN22^−/−^ CD44^+^ CD4 cells 8 dpi.

CD4 T cells and cytokines derived from these cells, such as IFN-γ and TNF, have pleiotropic antiviral effects and are essential for the control of viremia in LCMV Cl13 infection (23, 24). Consequently, we analyzed the effect of PTPN22 on antiviral CD4 T cell responses in the LCMV Cl13 system. The differences in pro-inflammatory cytokine production were even more pronounced in the antiviral CD4 T cell compartment, where we detected significantly higher frequency of IFN-γ, TNF, and IL-2-producing CD4 T cells in the absence of PTPN22 8 and 30 dpi (Figures 5A–D, and data not shown). In addition, T-BET but not LFA-1 or IRF4 expression levels were higher on CD4^+^CD44^+^ T cells from PTPN22^−/−^ mice as compared to controls 8 dpi (Figure 5E).

**Figure 6:**
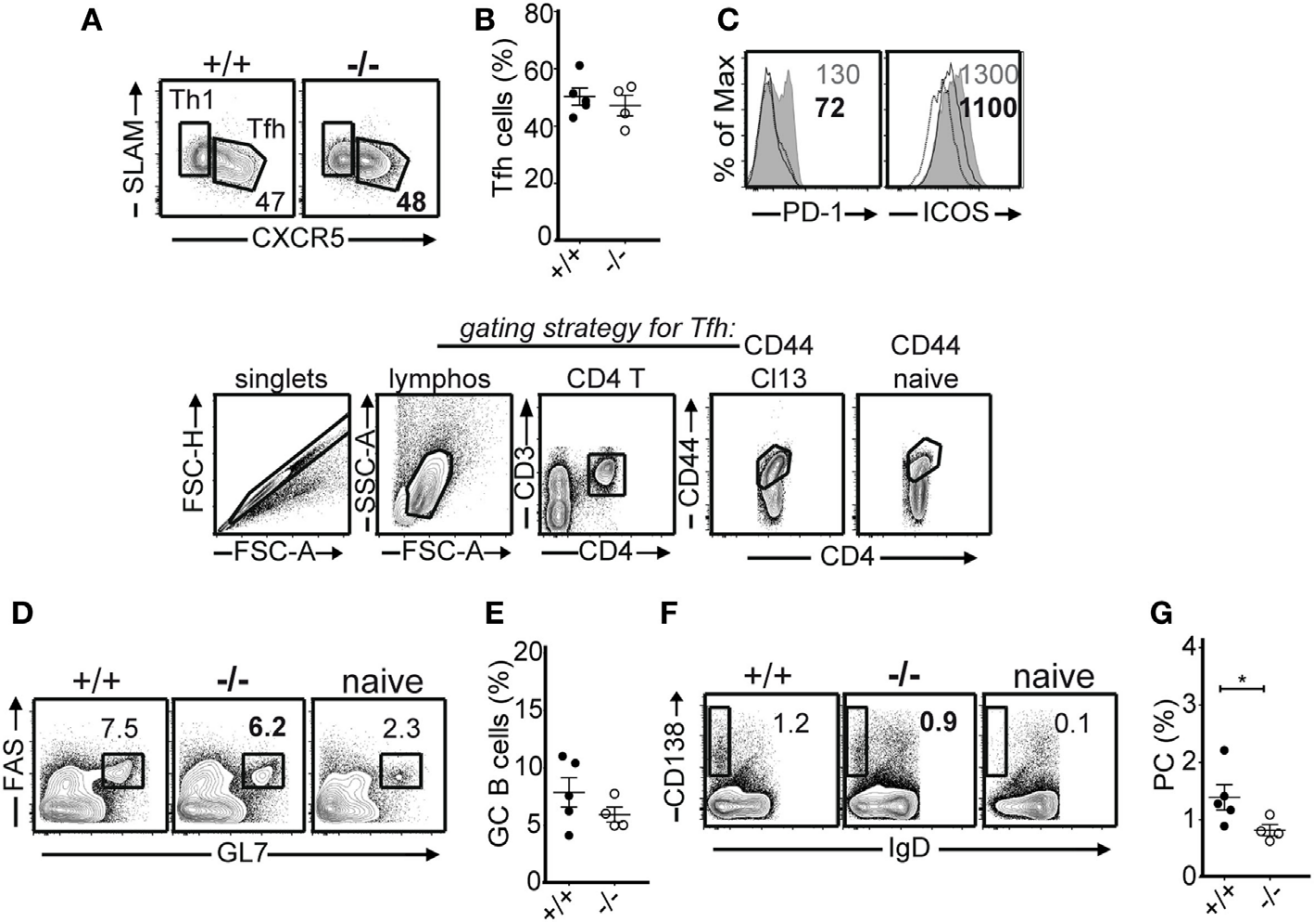
Similar germinal center responses in the absence of protein tyrosine phosphatase non-receptor 22 (PTPN22) in the lymphocytic choriomeningitis virus clone 13 infection system. **(A,B)** Representative FACS plots and frequency of T follicular helper (Tfh) (SLAM^lo^CXCR5^+^) and Th1 (SLAM^+^CXCR5^−^) splenic cells in PTPN22^+/+^ and PTPN22^−/−^ mice 30 days post infection (dpi) (gate: CD3^+^CD4^+^CD44^+^) are shown below. **(C)** Histogram overlay depicting PD-1 and ICOS expression levels (geometric mean) on Tfh cells from PTPN22^+/+^ (gray filled histogram) and PTPN22^−/−^ (black solid line). Naïve CD4 cells (black dotted line) were used as control. **(D,E)** Representative FACS plots and frequency of germinal center B cells (FAS^+^GL7^+^) in the spleen of PTPN22^+/+^ and PTPN22^−/−^ mice 30 dpi (gate: CD19^+^B220^+^). **(F,G)** Representative FACS plots and frequency of plasma cells (IgD^−^CD138^+^) in the spleen of PTPN22^+/+^ and PTPN22^−/−^ mice 30 dpi (gate: CD19^+^). Data are derived from at least two independent experiments, one to two experiments are shown per graph.

T follicular helper (Tfh) plays an important role in antiviral antibody responses to LCMV Cl13 infection (25, 26). While in LCMV acute infection the number of Tfh cells and the expression of Tfh effector molecules decrease after viral resolution, during persistent infection this does not happen due to the prolonged antigenic stimulation and a second wave of IL-6 production (25, 26). In the LCMV Arm acute infection system, we found no differences in Tfh development in the absence of PTPN22 (11). However, Maine et al. reported in two different studies that PTPN22 impacts the differentiation of Tfh (27, 28). Here, we examined the effects of PTPN22 on Tfh and germinal center (GC) responses after LCMV Cl13 infection. We did not observe differences in the frequency of Tfh (SLAM^lo^CXCR5^+^) between PTPN22^+/+^ and PTPN22^−/−^ mice 30 dpi (Figures 6A,B). Although PD-1 is not a suitable marker to determine GC Tfh cells in persistent infection as the majority of virus-specific CD4 cells express it at high levels, its expression levels along with those of ICOS were reduced on Tfh from PTPN22^−/−^ mice (Figure 6C). Tfh analyses at earlier time points, i.e., 8 dpi, showed similar results (not shown). The frequency of GC B cells was comparable 30 dpi (Figures 6D,E), whereas that of plasma cells was lower in PTPN22^−/−^ mice (Figures 6F,G). Together, these findings clearly show that PTPN22 inhibits pro-inflammatory virus-specific CD4 T cell responses during LCMV Cl13 infection.

### PTPN22 Promotes CTL Exhaustion through CD8 T Cell-Extrinsic Effects

**Figure 7:**
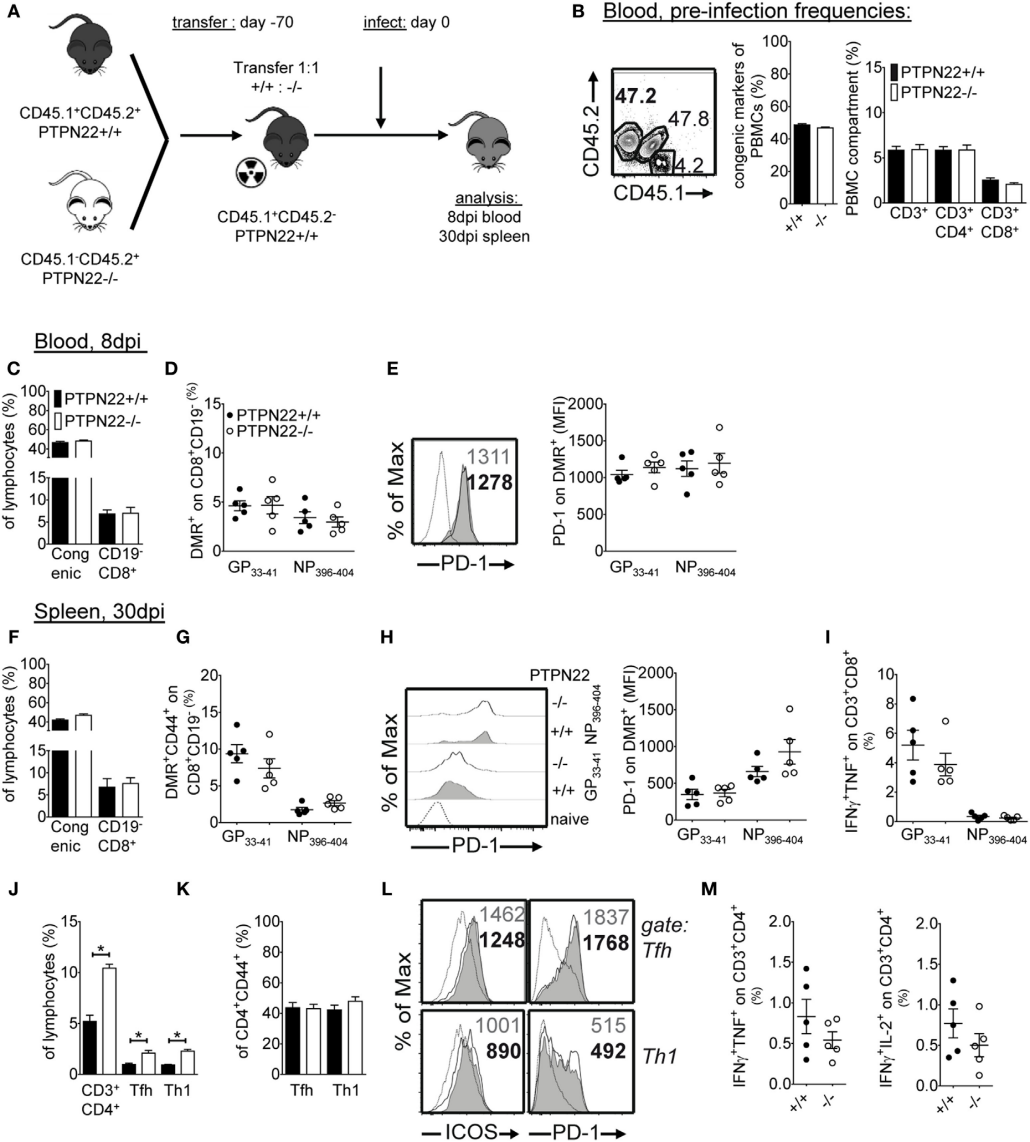
The improved CD8 T cell response of protein tyrosine phosphatase non-receptor 22 (PTPN22) deficiency to lymphocytic choriomeningitis virus clone 13 is T cell-extrinsic. **(A)** Schematic representation of the experimental outline. Lethally irradiated CD45.1^+^ mice were reconstituted with a 1:1 mixture of CD45.1^+^CD45.2^+^ PTPN22^+/+^:CD45.1^−^CD45.2^+^ PTPN22^−/−^ bone marrow cells. **(B)** At 60 days after reconstitution, blood was withdrawn to evaluate the proportion of PTPN22^+/+^ and PTPN22^−/−^ total, CD3, CD8, and CD4 cells prior to infection. **(C,D)** Mice were infected as in Figure 1. The proportions of CD3, CD8, and DMR^+^ cells were determined in the blood 8 days post infection (dpi). **(E)** Expression levels (geometric mean) of PD-1 were determined on DMR^+^ PTPN22^+/+^ and PTPN22^−/−^ CD8 T cells in the blood 8 dpi, with representative histogram overlay for GP_33–41_-specific cytotoxic lymphocytes shown. **(F,G)** The proportions of CD3, CD8, and DMR^+^ cells were determined in the spleen 30 dpi. **(H)** PD-1 expression levels (geometric mean) were determined on DMR^+^ PTPN22^+/+^ and PTPN22^−/−^ CD8 T cells in the spleen 30 dpi with representative histogram overlay shown. **(I)** The proportions of IFN-γ/TNF CD8 T cells were evaluated by intracellular cytokine staining following stimulation with GP_33–41_ and NP_396–404_ peptides [PTPN22^+/+^ (black circles), PTPN22^−/−^ (white circles)]. **(J)** The proportions of PTPN22^+/+^ and PTPN22^−/−^ CD4, Th1 (SLAM^+^CXCR5^−^) and T follicular helper (Tfh) (SLAM^lo^CXCR5^+^) T cells were determined 30 dpi in the spleen. **(K)** Frequencies of Th1 and Tfh PTPN22^+/+^ and PTPN22^−/−^ cells gated on CD4^+^CD44^+^ are shown. **(L)** Representative ICOS and PD-1 expression levels on PTPN22^+/+^ and PTPN22^−/−^ Th1 and Tfh cells are shown. **(M)** Splenocytes from chimeric mice were stimulated with GP_61–80_ peptide and stained for the indicated cytokines. Coproduction of IFN-γ/TNF and IFN-γ/IL-2 was evaluated [PTPN22^+/+^ (black circles), PTPN22^−/−^ (white circles)]. Data are representative of two independent mixed bone marrow chimera experiments. Number of symbols reflects number of mice per group.

The action of PTPN22 on T cells could be cell-autonomous or extrinsic, since PTPN22 has a direct role in T cells and in early innate pro-inflammatory responses (6, 10). To determine if CD8 T cell-intrinsic effects were responsible for improved viral titers in LCMV Cl13-infected mice, we performed mixed bone marrow chimeras. If the effect of PTPN22 on CD8 T cell exhaustion is T cell-extrinsic, then PTPN22^+/+^ and PTPN22^−/−^ CD8 T cells would be equally affected and their number and exhaustion phenotype would be similar. Instead, if the effect of PTPN22 is T cell-intrinsic, then PTPN22^−/−^ CD8 T cells would be at a competitive advantage and will be less exhausted. We reconstituted lethally irradiated C57BL/6 mice (CD45.1^+^CD45.2^−^) with 1:1 mixture of CD45.1^+^CD45.2^+^ PTPN22^+/+^: CD45.1^−^CD45.2^+^ PTPN22^−/−^ bone marrow cells (Figure 7A). For control, we used 1:1 mixture of CD45.1^+^CD45.2^+^ PTPN22^+/+^:CD45.1^−^CD45.2^+^ PTPN22^+/+^ bone marrow cells. Donors were traced by congenic markers and the CD45.1^+^CD45.2^+^:CD45.1^−^CD45.2^+^ ratio in peripheral blood was 1:1 and similar proportions of CD4 and CD8 T cells could be detected prior to infection (Figure 7B).

Eight days p.i. similar number of total circulating CD8 T cells between PTPN22^+/+^ and PTPN22^−/−^ lymphocytes were observed (Figure 7C). Among CD8 T cells, the proportion of DMR^+^ LCMV-specific CTLs was also similar (Figure 7D). Additionally, LCMV-specific CTLs displayed similar levels of the inhibitory receptor PD-1 (Figure 7E). At 30 dpi, there was still no significant effect of PTPN22 on total CD8 splenocytes, DMR^+^ CD8 T cells and PD-1 expression levels (Figures 7F–H). The frequency of cytokine-producing CTLs was also similar in the two PTPN22 compartments (Figure 7I), a finding which is in line with their evenly exhausted phenotype. Results in the control PTPN22^+/+^:PTPN22^+/+^ mixed bone marrow chimeras were similar (data not shown). Taken together, these results demonstrate that when PTPN22^−/−^ CTLs are found in the same environment as PTPN22^+/+^ CTLs are equally exhausted. Thus, the effect of PTPN22 on CD8 T cell exhaustion is CD8 T cell-extrinsic.

We additionally analyzed the CD4 compartment in the bone marrow chimera experiments. At 30 dpi, there was a significant effect of PTPN22 on total CD4 T cells, with more CD4 T cells in the PTPN22^−/−^ compartment (Figure 7J). Among CD4 T cells, there was a two-fold increase in PTPN22^−/−^ Tfh cells and Th1 cells (Figure 7J). However, this increase was due to the accumulation of the CD4 compartment and not due to a differentiation advantage of PTPN22^−/−^ CD4 T cells into Tfh (SLAM^lo^CXCR5^+^) or Th1 (SLAM^+^CXCR5^−^), since the frequency of PTPN22^+/+^ and PTPN22^−/−^ Tfh and Th1 within CD4s was similar (Figure 7K). Additionally, PD-1 and ICOS levels and cytokine production were similar (Figures 7L,M). These data demonstrate that under competitive conditions, PTPN22^−/−^ CD4 T cells show an expansion advantage over WT CD4 T cells but have similar potential in differentiating into Tfh and Th1. Hence, PTPN22 controls CD4 accumulation during LCMV Cl13 infection *via* T cell-intrinsic mechanisms.

## Discussion

The human variant of PTPN22 R619W is strongly associated with autoimmunity. While there have been a number of studies addressing the role of PTPN22 in different models of autoimmunity (3), relatively less has been done to address the role of PTPN22 in immunity against pathogens. PTPN22 is of particular interest in this regard because it plays a significant role in antigen receptor signaling in T and B cells [reviewed in Ref. (4)], as well in TLR signaling in innate cells (10). During chronic viral infection, persistent antigen presentation results in functional exhaustion of T cell responses, characterized by upregulation of inhibitory molecules and a progressive loss of effector T cell functions (29). Here, by studying the effect of PTPN22 in the LCMV Cl13 persistent infection system, we show that PTPN22^−/−^ mice clear the virus faster and retain higher CD8 and CD4 T cell functions. PTPN22 promotes CD8 T cell exhaustion by acting on CD8 T cell-extrinsic factors and inhibits CD4 T cell accumulation by acting on CD4 T cell-intrinsic factors. The CD8 T cell-extrinsic role of PTPN22 for control of LCMV Cl13 was demonstrated with mixed bone marrow experiments, where the exhausted phenotype of PTPN22^−/−^ CD8 T cells was identical to that of PTPN22^+/+^ CD8 T cells. In the same experiments, PTPN22^−/−^ CD4 T cells showed an accumulation advantage over the PTPN22^+/+^ CD4 T cells. Together, our results demonstrate an important role for PTPN22 that impacts postpriming accumulation of CD4 T cells and effector differentiation of CD4 and CD8 T cells.

Recently, a similar study was published by Professor Sherman’s group (30). Similar to ours, Maine et al. found that PTPN22-deficient mice show accelerated viral clearance of LCMV Cl13 infection by producing higher numbers of viral-specific CD4 and CD8 T lymphocytes with heightened function (30). PTPN22 deficiency did not result in a change in the number of GP_33–41_-specific LCMV-specific CTLs 8 dpi. However, at later time points (in our case 30 dpi), there was almost a twofold increase in the number of PTPN22^−/−^ GP_33–41_-specific CTLs, which displayed less exhausted phenotype and heightened functionality. We additionally analyzed the responses to other known LCMV-specific epitopes, such as the NP_396–404_, GP_276–286_ and NP_205–212_, and found them increased in PTPN22^−/−^ mice compared to the control groups. We also determined whether the effect of PTPN22 was T cell-extrinsic or determined by extrinsic factors by performing bone marrow chimeras. We found no change in the number or function of LCMV-specific CTLs 30 dpi, suggesting that PTPN22 promoted CD8 T cell exhaustion by T cell-extrinsic factors.

Maine et al. identified an important role of the augmented CD4 responses in viral protection. They showed no protection from viral persistence in PTPN22^−/−^ mice when CD4 T cells were depleted (30). Our bone marrow chimera studies demonstrated a critical role for PTPN22 in postpriming accumulation of CD4 T cells. Interestingly, some expansion advantage of CD4 T cells was also seen by Professor Sherman’s group using class II-restricted TCR transgenic T cells and adoptive transfer experiments (30). In their studies, Maine et al. also found that the resulting CD4 T cells produced higher levels of effector cytokines. As suggested by Maine et al., PTPN22 expression in CD4 T cells may promote the exhaustion phenotype of LCMV-specific CTLs.

We additionally analyzed the expression levels of T-BET, LFA-1, and IRF4 in CD4 and CD8 T cells. Whereas no differences were observed in CD8 T cells, T-BET expression levels were higher on CD4^+^CD44^+^ T cells from PTPN22^−/−^ mice 8 dpi, suggesting that PTPN22 dampens the pro-inflammatory function of CD4 T cells *via* T-BET downmodulation. Neutralizing antibodies against LCMV Cl13 appear at around 90 days after infection and are essential for viral clearance in WT mice (31). Both Maine et al. and our group studied GC responses and observed no significant differences, suggesting that the humoral response against LCMV was not involved in the enhanced viral clearance observed in PTPN22^−/−^ mice.

Innate immune responses to viruses critically impact the development of adaptive immune responses. Dendritic cells (DCs) play key role in the T cell response against LCMV Cl13 by producing higher levels of type I IFNs (32). In contrast to what was initially believed, however, type I IFNs exert both beneficial and detrimental effects on antiviral immunity (33). Type I IFNs not only promote innate and adaptive immune activation but are also responsible for the exhaustion of T cells (34, 35). Consequently, blocking IFNA receptor signaling promoted CTL functions and viral clearance (34, 35). IFNα and IFNβ play central but non-redundant role during LCMV Cl13 infection. However, solely IFNβ blockade could replicate the effect of IFNAR blockade (36). Thus, despite using the same receptor, IFNβ is responsible for promoting viral persistence. Previous studies showed that PTPN22 was needed for efficient activation and TLR-induced type I IFN in myeloid cells following acute infection with LCMV Arm (10). Maine et al. also found that plasmacytoid DCs from PTPN22^−/−^ mice infected with LCMV Cl13 produced lower levels of type I IFNs (30). Thus, in line with the proposed mechanism by Maine et al., the reduction in IFNβ production in PTPN22^−/−^ mice might contribute to the control of the LCMV Cl13 infection.

By utilizing the TCR transgenic system specific for the LCMV immunodominant epitope GP_33–41_ (P14) and adoptive transfer experiments, we previously showed that P14.PTPN22^−/−^ CD8 T cells fail to expand in PTPN22-sufficient hosts following infection with the acute viral strain of LCMV (Arm) due to T cell-intrinsic defects in IFNAR signaling (12). Here, we show that PTPN22 promotes CTL exhaustion in the LCMV Cl13 system *via* T cell-extrinsic factors. Collectively, these findings suggest that PTPN22 not only supports CD8 T cell accumulation *via* T cell-intrinsic mechanisms but also promotes T cell exhaustion by acting on T cell-extrinsic factors. This dual effect of PTPN22 is likely a mechanism that serves to prevent excessive immunopathology and mortality.

Taken together, we report a critical role for PTPN22 in the control of chronic LCMV Cl13 infection. We show that PTPN22 controls the exhaustion phenotype of both CD4 and CD8 T cells and that PTPN22^−/−^ mice have improved CD4 and CD8 T cell responses and increased viral control. Furthermore, we demonstrate that the effects of PTPN22 on CD8 T cell exhaustion phenotype are T cell-extrinsic. Our data suggest that if PTPN22 R620W is a loss-of-function allele, carriers would be more resistant to chronic infections as compared to controls. These studies identify PTPN22 as a promising therapeutic target for chronic infection.

## Ethics Statement

Mice were housed under spf conditions in compliance with guidelines of the San Raffaele Institutional Animal Care and Use Committee (IACUC approved protocol #677).

## Author Contributions

TJ, GG, RF, and BM performed experiments and analyzed data; MK performed experiments, analyzed data, and revised the manuscript, MI and MB discussed data; SS-A discussed data and revised manuscript; GF designed and performed experiments, analyzed data, and wrote the manuscript. All the authors have read and approved the manuscript.

## Conflict of Interest Statement

The research was conducted in the absence of any commercial or financial relationships that could be construed as a potential conflict of interest.

## Abbreviations

PTPN22, protein tyrosine phosphatase non-receptor 22; LCMV Arm, lymphocytic choriomeningitis virus Armstrong; LCMV Cl13, lymphocytic choriomeningitis virus clone 13; CTL, cytotoxic lymphocyte; DMR, dextramer; ICCS, intracellular cytokine staining; Tfh, T follicular helper; GC, germinal center; DC, dendritic cell; dpi, days post infection; spf, specific pathogen free.
